# Burden of sleep disturbances and associated risk factors: A cross-sectional survey among HIV-infected persons on antiretroviral therapy across China

**DOI:** 10.1038/s41598-017-03968-3

**Published:** 2017-06-16

**Authors:** Xiaojie Huang, Huiqin Li, Kathrine Meyers, Wei Xia, Zhihao Meng, Chongxi Li, Jinsong Bai, Shenghua He, Weiping Cai, Chengyu Huang, Shuiqing Liu, Hui Wang, Xuemei Ling, Ping Ma, Daling Tan, Fuxiang Wang, Lianguo Ruan, Hongxin Zhao, Hongxia Wei, Yanfen Liu, Jianhua Yu, Hongzhou Lu, Min Wang, Tong Zhang, Hui Chen, Hao Wu

**Affiliations:** 1 Center for Infectious Diseases, Beijing You’an Hospital, Capital Medical University, Beijing, 100069 China; 2 Infectious Diseases Department, Yunnan AIDS Caring Center, Kunming, Yunnan 650301 China; 3 The Aaron Diamond AIDS Research Center, New York, NY 10016 United States; 4 Infectious Diseases Department, Longtan Hospital of Guangxi, Liuzhou, Guangxi 545005 China; 5 Infectious Diseases Department, the Third People’s Hospital of Kunming, Kunming, Yunnan 650041 China; 6 Infectious Diseases Department, the Infectious Diseases Hospital of Chengdu, Chengdu, Sichuan 610061 China; 7 Institute of Infectious Diseases, the Eighth People’s Hospital of Guangzhou, Guangzhou, Guangdong 510060 China; 8 Department of AIDS, Chongqing Infectious Disease Medical Center, Chongqing, 400039 China; 9 Department of Infectious Diseases, the Fifth People’s Hospital of Guiyang, Guiyang, Guizhou 550004 China; 10 Department of Clinical AIDS Research, the Third People’s Hospital of Shenzhen, Shenzhen, Guangdong 518020 China; 11 Department of Hematology, the Third People’s Hospital of Hengyang, Hengyang, Hunan 421000 China; 12 Department of Infectious Diseases, the Second People’s Hospital of Tianjin, Tianjin, 300193 China; 13 Department of Infectious Diseases, the People’s Hospital of Luzhai, Liuzhou, Guangxi 545600 China; 14 Department of Infectious Diseases, the First Affiliated Hospital of Harbin Medical University, Harbin, Heilongjiang 150001 China; 15 Department of Infectious Diseases, Wuhan Medical Treatment Center, Wuhan, Hubei 430023 China; 16 Department of Infectious Diseases, Beijing Ditan Hospital, Capital Medical University, Beijing, 100015 China; 17 Department of Infectious Diseases, the Second Hospital of Nanjing Affiliated to Southeast University Medical College, Nanjing, Jiangsu 210003 China; 18 Department of Infectious Diseases, the Fourth People’s Hospital of Nanning, Nanning, Guangxi 530023 China; 19 Department of Infectious Diseases, the Sixth People’s Hospital of Hangzhou, Hangzhou, Jiangsu 310023 China; 20 Department of Infectious Diseases, Shanghai Public Health Clinical Center Affiliated to Fudan University, Shanghai, 200083 China; 21 Department of Infectious Diseases, the First People’s Hospital of Changsha, Changsha, Hunan 410005 China; 22 School of Biomedical Engineering, Capital Medical University, Beijing, 100069 China

## Abstract

This study evaluated the prevalence and factors associated with sleep disturbance in a large cohort of HIV-infected patients across China. A cross-sectional study was conducted among HIV-infected patients on antiretroviral therapy at 20 AIDS clinics. The Pittsburgh Sleep Quality Index was self-administered by subjects. Socio-demographic characteristics, medical history and HIV-related clinical data were collected. 4103 patients had complete data for analysis. Sleep disturbances were observed in 43.1% of patients. Associated factors in multivariable analysis included psychological factors: anxiety (odds ratio [OR], 3.13; 95% confidence interval [CI], 2.44–4.00; P < 0.001), depression (OR, 2.09; 95% CI, 1.70–2.57; P < 0.001), and both anxiety and depression (OR, 5.90; 95% CI, 4.86–7.16; P < 0.001); sociodemographic factors: MSM (OR, 1.26; 95% CI, 1.04–1.52; P﻿ = 0.018), being single (OR, 1.45; 95%CI 1.21–1.74; P < 0.001), higher education (OR, ﻿1.25; 95% CI, 1.03–1.53; P = 0.025)﻿; and clinical factors: suboptimal adherence (OR,1.51; 95% CI,1.23–1.85; P < 0.001), regimen-switching (OR, 1.94; 95% CI, 1.12–3.35; P = 0.018), and antidepressant use (OR, 1.98; 95% CI, 1.47–2.67; P = 0.044). Prevalence of sleep disturbance is high in this large Chinese cohort. Associated factors appear related to psychological and social-demographic factors. Health workers may consider routinely assessing sleep disturbances among HIV-infected patients, especially in the first three months after HIV diagnosis, and referring for mental health services, which may positively impact adherence to treatment.

## Introduction

HIV-infected individuals appear to be more vulnerable to sleep disturbances than the general population^1–4^. However, awareness of sleep disturbance as a health issue in general and as an HIV-related health issue specifically is low among patients, who do not emphasize such issues with their physician^5, 6^. Administration of standardized sleep assessment tools, such as the Pittsburg Sleep Quality Index (PSQI), Diagnostic and Statistical Manual of Mental Disorders (DSM-IV), and the International Classification of Sleep Disorders (ICSD-2) in cohorts of HIV-infected individuals have observed a high prevalence of sleep disturbances from 40% to 70%^4, 7, 8^. Major sleep disturbances frequently go undiagnosed and untreated and may have severe health consequences, such as depression and anxiety^6, 9^. A range of primary sleep disorder symptoms overlap with psychological and behavioral factors, requiring targeted diagnostic and treatment interventions^10, 11^.

The pathophysiology of sleeping disturbances among HIV-infected patients is unclear, but may be related to the ability of HIV to infect the central nervous system (CNS), the impact of antiretroviral medications, mental health issues, and substance abuse^6, 12–14^. Sleep disturbances occur throughout all stages of infection, and may be associated with the virus itself, antiretroviral drugs, or antidepressants. It may also increase the risk for psychiatric disorders, cardiovascular morbidity, and mortality, but the degree and direction of causality are unclear^15, 16^.

Although the prevalence of sleeping disorders is reportedly high among HIV-infected persons, relatively few studies exist in the literature during the antiretroviral therapy (ART) era^15, 17^. Recent studies have largely focused on the impact of specific antiretroviral agents (e.g., efavirenz) on sleep disturbances^18–20^. Anxiety and depression are psychological factors that have been found to affect sleep quality^4, 21, 22^. Regardless of its etiology, sleep disturbances is clinically important in this population due to potential impact on quality of life, adherence to ART cognition impairment, comorbid psychiatric disorders like anxiety/depression and immune system function^6, 23, 24^. The purpose of this paper is to evaluate the epidemiology and associated risk factors for sleep disorders in AIDS patients and to recommend early evaluation for and detection of these conditions among people living with HIV.

## Results

### Characteristics of study population

We evaluated 4103 HIV-infected persons (Tables 1 and 2). Mean age of the cohort was 37.6 years (standard deviation [SD], 11.7 years), 79.4% were male, and 38.5% were MSM. The median time from HIV diagnosis to study enrollment was 27 months (interquartile range [IQR], 11–58 months), the median duration of ART was 18 months (IQR, 6–43 months), 15.5% had a current CD4+ cell count below 200 cells/mm3, and 48.4% had an undetectable HIV RNA level. 75.4% of patients were on their original first-line ART regimen and had not experienced an ART regimen switch.

**Table 1:** Demographic characteristics for all 4103 subjects [n (%)].

Characteristics	Levels				
Age, years	<30	30–50	>50	Missing	
	1175 (28.6%)	2306 (56.2%)	569 (13.9%)	53 (1.3%)	
Sex	Male	Female	Missing		
	3204 (78.1%)	831 (20.3%)	68 (1.7%)		
Education	<High school	High school	>High school	Missing	
	1528 (37.2%)	988 (24.1%)	1527 (37.2%)	60 (1.5%)	
Transmission route	Heterosexual	Homosexual/bisexual	Blood transfusion	PWID	Others
	1589 (38.9%)	1730 (42.2%)	39 (1.0%)	216 (5.3%)	520 (12.7%)
Marital status	Married	Divorced	Widowed	Single	Missing
	1698 (41.4%)	456 (11.1%)	132 (3.2%)	1718 (41.9%)	99 (2.4%)
Employment	White collar	Blue collar	Unemployed	Student	
	1158 (28.2%)	1835 (44.7%)	280 (6.8%)	830 (20.2%)	
Family history of mental illness	Yes	No	Missing		
	85 (2.1%)	4004 (97.6%)	14 (0.3%)		
Personal history of mental illness	Yes	No	Missing		
	65 (1.6%)	4026 (98.1%)	12 (0.3%)		
Current sleep medication use	Yes	No			
	236 (5.8%)	3867 (94.2%)			
Current antidepressant use	Yes	No	Missing		
	197 (4.8%)	3884 (94.7%)	22 (0.5%)		
HIV status disclosed to friends	Yes	No	Missing		
	1092 (26.6%)	2996 (73.0%)	15 (0.4%)		
HIV status disclosed to family	Yes	No	Missing		
	2464 (60.1%)	1628 (38.7%)	11 (0.3%)		
Support from family/friends	Yes	No	Missing		
	2803 (68.3%)	1239 (30.2%)	61 (1.5%)

**Table 2:** Clinical characteristics for all 4103 subjects [n (%)].

Characteristics	Levels				
Current CD4^+^ cell count, cells/mm^3^	<50	50–200	200–500	≥500	Missing
	128 (3.1%)	506 (12.3%)	2160 (52.6%)	1272 (31.0%)	37 (0.9%)
Current viral load	Undetectable	Detectable	Missing		
	1672 (40.8%)	1781 (43.4%)	650 (15.8%)		
Time since HIV diagnosis	<3 months	≥3 months	Missing		
	297 (7.2%)	374 (91.3%)	58 (1.4%)		
Duration of ART	<1 year	≥1 year	Missing		
	1514 (36.9%)	2528 (61.6%)	61 (1.5%)		
Current EFV-based regimen	Yes	No			
	2944 (71.8%)	1159 (28.2%)			
Duration of EFV exposure	0 month	<3 months	≥3 months	Missing	
	1159 (28.2%)	681 (16.6%)	2217 (54.0%)	46 (1.1%)	
Drug Adherence	≥95%	<95%			
	3642 (88.8%)	461 (11.2%)			
Regimen switching	First-line ART regimen	Switched ART			
	3093 (75.4%)	1010 (24.6%)			
Current ART regimen pill burden	3	>3			
	2438 (59.4%)	1665 (40.6%)			
Number of ART Regimens	0	1	2	3	
	3067 (74.8%)	726 (17.7%)	233 (5.7%)	77 (1.9%)	

### Prevalence of sleep disturbances

Median PSQI score for 3873 HIV-infected persons was 5 (IQR: 3–7). Over 40% (43.1%, 95% confidence interval [CI], 41.5%–44.7%) of participants had a PSQI score >5, suggestive of sleep disturbance.

### Prevalence of sleep disturbances among patients with anxiety and depression

Median HADS score was 5 (IQR: 2–8) for anxiety and 5 (IQR: 2–9) for depression. Among the 3873 HIV-infected persons evaluated for anxiety and depression, prevalence of sleep disturbances differed significantly (P < 0.001) between those who suffered anxiety (68.1%) and those who did not (33.6%), and between those who suffered depression (61.2%) and those who did not (34.3%).

### Factors associated with sleep disturbances

In univariate analyses, factors associated with sleep disturbance (PSQI > 5) included age, education, transmission route, marital status, support from family/friends, time of diagnosed with HIV infection, drug adherence, number of ART regimens, current antidepressant use, and anxiety and depression. There were no significant associations with HIV-specific clinical factors including ART use (including EFV- containing regimens) and sleep. In the final multivariable model combining all of the significant variables above, factors associated with sleep disturbance based on the PSQI included education (P = 0.044), transmission route (P = 0.040), marital status (P < 0.001), drug adherence (P = 0.001), current EFV-based regimen (P = 0.039), antidepressant use (P = 0.039), and anxiety and depression (P < 0.001) (Table 3).

**Table 3:** Demographic and clinical factors associated with sleep disturbances.

Characteristics	% of subjects with PSQI > 5	Univariate Model		Multivariable Model	
		OR (95% CI)	P Value	OR (95% CI)	P Value
DEMOGRAPHIC FACTORS					
Age, years			0.035		
<30	45.1%	1.32 (1.07–1.63)	0.010		
30–50	43.3%	1.22 (1.01–1.49)	0.041		
>50	38.4%	1			
Gender			0.633		
Female	42.3%	1			
Male	43.3%	1.04 (0.89–1.22)			
Education			0.003		0.046
<High school	39.6%	1		1	
High school	44.3%	1.22 (1.03–1.44)	0.022	1.22 (1.01–1.48)	0.039
>High school	39.6%	1.29 (1.11–1.49)	0.001	1.25 (1.03–1.53)	0.025
Transmission route			<0.001		0.050
Heterosexual	37.5%	1		1	
Homosexual/bisexual	46.5%	1.45 (1.26–1.67)	<0.001	1.26 (1.04–1.52)	0.018
Blood transfusion	40.0%	1.11 (0.56–2.20)	0.761	0.99 (0.46–2.14)	0.988
PWID	53.7%	1.93 (1.44–2.59)	<0.001	1.40 (0.99–1.97)	0.056
Others	45.1%	1.37 (1.12–1.69)	0.003	1.31 (1.04–1.65)	0.024
Marital status			<0.001		0.001
Married	37.7%	1		1	
Divorced	45.7%	1.39 (1.12–1.73)	0.003	1.24 (0.98–1.58)	0.074
Widowed	46.3%	1.42 (0.98–2.07)	0.062	1.53 (1.01–2.33)	0.046
Single	47.5%	1.50 (1.30–1.72)	<0.001	1.45 (1.21–1.74)	<0.001
Employment			0.097		
White collar	43.7%	1			
Blue collar	41.7%	0.92 (0.79–1.07)	0.284		
Unemployed	40.0%	0.86 (0.65–1.13)	0.273		
Student	46.5%	1.12 (0.93–1.34)	0.233		
Support from family/friends			<0.001		
Yes	41.2%	1			
No	47.5%	1.29 (1.13–1.48)			
CLINICAL FACTORS					
Current CD4^+^ cell count, cells/mm3			0.870		
<50	46.7%	1.17 (0.81–1.71)	0.402		
50–199	43.3%	1.02 (0.83–1.270	0.834		
200–499	43.1%	1.01 (0.88–1.17)	0.864		
≥500	42.8%	1			
Current viral load			0.981		
Undetectable	43.1%	1			
Detectable	44.5%	1.00 (0.87–1.15)			
Time since HIV diagnosis			0.035		
<3 months	49.3%	1.30 (1.02–1.67)			
≥3 months	42.7%	1			
Drug adherence			<0.001		0.002
≥95%	42.0%	1		1	
<95%	52.2%	1.51 (1.23–1.85)		1.45 (1.14–1.83)	
Current EFV-based regimen			0.331		
Yes	42.7%	1			
No	44.4%	1.07 (0.93–1.24)			
Duration of EFV exposure			0.141		
0 month	44.4%	1.12 (0.97–1.30)	0.137		
<3 months	45.4%	1.17 (0.98–1.39)	0.093		
≥3months	41.6%	1			
Duration of ART			0.127		
<1 year	44.7%	1.11 (0.97–1.27)			
≥1 year	42.2%	1			
Number of ART Regimens			0.019		0.045
0	42.3%	1		1	
1	43.0%	1.03 (0.87–1.21)	0.763	1.11 (0.92–1.35)	0.278
2	49.6%	1.34 (1.02–1.76)	0.036	1.27 (0.94–1.73)	0.126
3	57.1%	1.82 (1.12–2.93)	0.015	1.94 (1.12–3.35)	0.018
Antidepressant use			<0.001		0.044
Yes	22.6%	1		1.98 (1.47–2.67)	
No	42.3%	1.98 (1.47–2.67)		1	
Comorbidities			<0.001		<0.001
Anxiety < 8 & Depression < 8	30.7%	1		1	
Anxiety < 8 & Depression ≥ 8	46.0%	1.93 (1.59–2.33)	<0.001	2.09 (1.70–2.57)	<0.001
Anxiety ≥ 8 & Depression < 8	59.4%	3.30 (2.60–4.18)	<0.001	3.13 (2.44–4.00)	<0.001
Anxiety ≥ 8 & Depression ≥ 8	72.0%	5.81 (4.86–6.98)	<0.001	5.90 (4.86–7.16)	<0.001

Regarding the association of poor sleep and depression by Hospital Anxiety and Depression (HAD) scores, sleep disturbance was present in 34.3% of those without depression (P < 0.001). Moreover, higher HAD scores were significantly associated with higher PSQI scores (r = 0.365; P < 0.001). Participants with a higher risk of anxiety (HAD > 8) had a 4-fold higher odds of sleep disturbance than those with lower HAD scores (HAD ≤ 8) (odds ratio [OR], 4.22; 95% CI, 3.63–4.91; P < 0.001), whereas those with a higher risk of depression (HAD > 8) had a 3-fold higher odds of sleep disturbance relative to those with a HAD ≤ 8 (OR, 3.01; 95% CI, 2.62–3.46; P < 0.001).

## Discussion

This study represents one of the largest epidemiologic studies of the prevalence of sleep disturbances as measured by PSQI among HIV-infected persons in the ART era^25, 26^. The PSQI is a standardized questionnaire frequently used in studies of HIV-positive persons, which has a cut-off score indicative of sleep disturbance, however, it lacks specificity for insomnia^11, 27–30^.

HIV remains a highly stigmatized condition in China, with patients reporting emotional, financial, and physical burdens resulting from stigma^31^. Discrimination is experienced from both medical and non-medical sources^31, 32^, despite legal protection from discrimination in accessing medical care, employment, and educational opportunities^33^. The potential interaction between the experience of stigma and sleep disturbance have not been studied in this context but could potentially play a role in understanding sleep disturbance in this population.

Data from general population surveys in China suggest that the prevalence of sleep disturbance in healthy populations is about 10%^34^. Our finding that HIV-infected persons have a high prevalence of sleep disturbance even in the ART era suggests that there are specific mechanisms causing poor sleep quality that are unique to this population or to HIV infection. HIV-infected persons with poor immune recovery did not have a statistically significantly higher rate of sleep disturbance when compared to those who had better immune recovery with nearly normal CD4^+^ cell counts above 500 cells/mm^3^. We did not find a relationship between sleep and CD4^+^ cell counts or HIV RNA in either direction. This is consistent with some studies that have not found any relationship^15, 18^, however others have found that sleep disturbances are independently related to immune status^26, 35–37^. Other studies conducted in the pre-ART era showed an association between sleep and CD4^+^ cell counts or HIV RNA^7, 38, 39^. The divergence of findings may be due to the differences in the tools used or to the fact that the prior studies were conducted in the pre-ART era when HIV perhaps played a more direct role in sleep.

Regarding HIV-specific factors, we found pill burden, poor adherence and more frequent switching of ART regimens were associated with higher proportions of poor sleep. These data suggest that in the ART era, patients with optimal adherence and patients who had not switched ART had lower rates of sleep disturbances. Duration of ART did not have statistical significance. Furthermore, patients on EFV-based regimen did not suffer a higher rate of sleep disturbance compared with patients on non-EFV-based regimens. However, patients who had initiated an EFV-based regimen in the last half year did have a marginally higher rate of sleep disturbance. This suggests that the sample may be biased, in that patients who had suffered CNS side effects from EFV may have switched regimens prior to the cross-sectional study, leaving only patients who did not suffer EFV-related side effects in the sample. In addition, antiretroviral agents with high CNS penetration effectiveness scores may be protective of HIV’s effects on the CNS. On the other hand, some drugs may be associated with adverse sleep effects (e.g., EFV), especially at higher plasma levels^36, 40, 41^. Data from prior studies among HIV-infected persons with EFV-based regimen did find much higher rates of sleep disturbance^40^. However, these studies were conducted when patients had concurrent opportunistic infections^42^, making attribution to EFV difficult. Our population consisted of all HIV-infected persons with free access to antiretroviral therapy, without AIDS-related opportunistic infections at the time of investigation.

In our study, the length of time since HIV diagnosis was found to be associated with sleep disturbances, with a significant association between shorter duration from diagnosis and poor quality of sleep. HIV infection remains a highly stigmatized illness in China, and can be accompanied by identity stigma and low self-esteem. These symptoms can be considered normal emotional responses to the reality of living with HIV as well as symptoms of depression. These may explain the higher rate of sleep disturbances in the first several months after diagnosis^7^. This finding however conflicts with that of other studies that have assessed sleep disturbances in relation to duration of infection. Imeri and Opp and Seay *et al*. did not find any association between them in their studies^39, 43^.

In addition, HIV-infected women did not have a statistically significantly burden of sleep disturbance compared with men. People who used illicit drugs and MSM had a higher rate of sleep disturbances as compared to heterosexuals, which may be related to their marginalized position in Chinese society^44^. Individuals with higher education had statistically greater odds of poor sleep compared with those with less education. Married individuals had a lower rate of sleep disturbance, potentially due to marriage conferring more family support, as we found that patients who report support from family or friends consistently had a lower rate of poor sleep.

The strongest associations found in our study were between depression and anxiety and sleep disturbance. This finding is consistent with other studies^21, 45, 46^, and shows that psychiatric disorders are a major factor in sleep disturbances among HIV-infected patients. Given the likely bidirectional association between sleep and depression or anxiety, targeted management of one may improve the other. Therefore, treating depression and anxiety might improve sleep quality, and addressing sleep disturbances may relieve psychological morbidity^45, 47, 48^.

Despite a high prevalence of sleep disturbance among HIV- infected persons, among patients with PSQI > 5, only 11.19% were regularly using sleep medication. Treatment with cognitive behavioral therapy for sleep disturbance or insomnia, considered first line treatment for insomnia, was not measured. Prior studies have also noted that most patients with sleep disturbances remain untreated and often have a poor understanding of available treatment options^8, 49^.

Our study had several potential limitations. Sleep disturbances were diagnosed based on questionnaire data rather than polysomnograms or actigraphy data; however, we used standardized instruments comparable to those used in other studies, and some experts suggest that self-reported data may be more representative of sleep issues^4, 27, 50^. The cross-sectional study design did not allow for assessment of the temporal relationship between poor sleep and factors (e.g., depression improvement or pre- and post-ART), the direction of causality, or for the assessment of whether sleep disturbances were transient or chronic in nature. Presence of pain, which has been shown to be associated with sleep disturbance in the literature, was not assessed. Finally, the lack of HIV-uninfected controls is a limitation of the study.

Our study had several strengths. This study is one of the largest study to date assessing sleep disturbance among HIV-infected patients on treatment^15, 38, 51^. It used validated measures that are quick and inexpensive to implement to assess sleep disturbances, anxiety and depression and could easily be integrated into routine clinical practice^27, 50^.

In summary, the high prevalence of poor sleep suggests that treating sleep disturbances in those with HIV infection may have a positive impact on their quality of life and could potentially yield improvements to adherence to ARTs and mental health. HIV providers may consider routinely assessing sleep disturbances especially in the first three months of HIV diagnosis, and referring for mental health and social support services if PSQI > 5. Therefore, integrating assessment of sleep disturbance into routine care and even extensive sleep quality evaluations at home for HIV-infected patients may be advisable in China. Prospective cohort studies that track sleep disturbance from time of diagnosis through treatment initiation and continuation could be useful to characterize the relationship between sleep disturbance, HIV, and treatment further.

## Methods

### Study Design and Participants

We conducted a cross-sectional study among 4724 HIV-infected adults on treatment, collecting data between January 2014 and December 2015 from patients at 20 HIV clinics across China covering Beijing, Guangxi, Yunnan *and other major municipalities*. The study enrolled patients accessing services at HIV clinics who were at least 18 years, diagnosed with HIV infection, on antiretroviral therapy, and not pregnant within the prior 3 months. Exclusion criteria included the presence of any acute medical condition that could affect the ability to complete the study questionnaire.

All participants provided written informed consent to complete a survey and have their medical data abstracted from their medical records. The study was approved by Beijing Youan Hospital institutional review board. All experiments were performed in accordance with relevant guidelines and regulations.

### Main Outcomes

Each participant completed the PSQI questionnaire, a 19-item questionnaire that assesses seven sleep components (sleep quality, sleep latency, sleep duration, habitual sleep efficiency, sleep disturbances, use of hypnotics, and daytime dysfunction) during the prior month. The scores for these components range from 0 (no difficulty) to 3 (severe difficulty) and are summed to produce a global measure of sleep disturbance, with a higher score denoting poorer sleep quality (range: 0–21). The Chinese version of PSQI was developed, widely used in adolescents, adults and elderly healthy and clinical groups, and showed good internal consistency, split half and retest reliability^52, 53^. We used the PSQI cut off of global score >5 on the PSQI instrument^27^ (sensitivity of 90% and specificity of 87%) to define sleep disturbance in this cohort. The HAD scale which consists of seven items relating to depression (HAD-D) and another seven relating to anxiety (HAD-A) was also administered and anxiety and depression were defined as a HAD score ≥8 on each subscale^54^.

Demographic, behavioral, and psychosocial data were collected through self-administered survey. Clinical data were abstracted from medical records. This included HIV-specific data, like date of HIV diagnosis, history of medical conditions, current CD4^+^ cell counts and HIV RNA levels, and ART regimen at the time of survey. 4103 (86.9%) patients had complete data for analysis.

### Statistical Analysis

Descriptive statistics are presented as means with SDs, or counts with proportions, as appropriate. Two sample t tests were used to compare means, and χ^2^ tests were used to compare proportions. Logistic regression analysis was used to investigate associations between sleep outcome and demographic, behavioral, psychosocial, and clinical factors. Odds ratios for sleep disturbance were estimated with 95% CIs. Factors with a P value < 0.10 in univariate models were initially included in the multivariable model and were then eliminated using backward selection. Linear regression and the Pearson correlation coefficient were used to explore the relationship between the PSQI score and HAD score. All P values were 2-sided, and P values < 0.05 were considered significant. Analyses were conducted using SPSS software 21.0.
